# The Independent and Combined Roles of Attentional and Interpretative Biases in Antisocial Behavior, Trait Aggression and Aggressive Responses Under Provocation

**DOI:** 10.1002/ab.70074

**Published:** 2026-06-14

**Authors:** Ignazio Puzzo, Anam Hamid‐Saifullah, Simona Cantarella, Anna Fieldwalker, Luke Aldridge‐Waddon, Veena Kumari

**Affiliations:** ^1^ University of Reading Reading UK; ^2^ Great Ormond Street Hospital for Children NHS Foundation Trust London UK; ^3^ Brunel University of London London UK

## Abstract

Aggressive behavior has been linked to biases in social information processing, including heightened attention to threat and hostile interpretations of ambiguous social cues. The extent to which attention and interpretational biases uniquely or jointly contribute to aggression under provocation remains unclear. The present study investigated the effects of attentional bias toward angry faces and hostile interpretation bias on aggression using both self‐report and behavioral measures in a non‐clinical sample. Participants (*n* = 67) completed an emotional attentional blink task and a hostile interpretation bias measure, alongside self‐report indices of aggression and antisocial behavior, and an experimental measure of provoked aggression. Hostile interpretation bias was positively associated with self‐reported aggression and antisocial tendencies, whereas attentional bias toward angry faces was selectively associated with behavioral aggression. Critically, these cognitive biases were examined as potentially independent, overlapping, or interactive contributors to aggression under provocation, and attentional and interpretation biases interacted to predict aggressive behavior under conditions of high provocation, such that individuals high in both biases displayed the greatest levels of aggression. No interaction effects were observed for self‐reported outcomes. These findings support social information‐processing models proposing that multiple cognitive biases jointly contribute to aggression and highlight the importance of considering situational provocation and outcome modality when examining cognitive risk factors for aggressive behavior and preventive cognitive interventions in at risk populations.

## 1 Introduction

Aggressive and antisocial behaviors represent a significant public health concern due to their association with interpersonal harm, criminal justice involvement, and long‐term psychosocial impairment (Chow et al. 2024; Home Office 2023). Aggression, defined as behavior intended to harm others, and antisocial behavior, which encompasses a broader pattern of rule‐breaking and norm‐violating actions, represent distinct but related constructs. A key aim of the present study is to examine whether attentional and interpretational biases operate as independent, overlapping, or interactive cognitive mechanisms contributing to these behaviors.

Cognitive theories of aggression emphasize biased social information processing as a proximal mechanism through which aggression is elicited, particularly in ambiguous or provocative contexts (Crick and Dodge 1994, 1996). Distortions in how individuals attend to and interpret social cues are theorized to be associated with increased perceived threat and a greater likelihood of retaliatory responding.

However, although attentional and interpretative biases are theoretically linked within social–cognitive models, they are typically studied in isolation. This limits understanding of whether they represent independent pathways to aggression or mutually reinforcing mechanisms that increase risk when co‐occurring. Clarifying their joint contribution is therefore important for refining contemporary models of aggression.

The Social Information Processing (SIP) model (Crick and Dodge 1994, 1996) provides a key framework for understanding cognitive mechanisms underlying aggression. It proposes that behavior emerges through sequential stages: encoding social cues, interpreting intent, generating responses, and selecting a behavioral outcome. Biases at early stages, particularly encoding and interpretation, can increase the likelihood of aggressive responding (Dodge et al. 2015). A central implication is that attentional and interpretative processes are dynamically linked, yet this integration has rarely been tested empirically. Consequently, it remains unclear whether attentional and interpretation biases exert additive, overlapping, or synergistic effects on aggression.

Attentional bias toward threat‐relevant stimuli, such as angry faces, reflects preferential allocation of perceptual resources to cues signaling interpersonal threat (Öhman et al. 2001). Neurocognitively, exaggerated capture by angry faces may reflect heightened reactivity in threat‐processing systems implicated in reactive aggression (Blair 2016). Consistent with this, individuals high in trait aggression or hostility show enhanced attentional capture by angry faces across behavioral and neurophysiological paradigms (Crago et al. 2019; Wilkowski et al. 2015). However, associations between attentional bias and aggression are typically modest, inconsistent and context dependent (Cooper and Langton 2006; Wilkowski et al. 2015).

One explanation concerns the psychometric limitations of commonly used reaction‐time indices, particularly dot‐probe difference scores, which frequently demonstrate low reliability and poor test–retest stability (Schmukle 2005; Kappenman et al. 2014). Measurement attenuation may therefore obscure true associations with individual differences in aggression. These limitations have prompted the use of alternative paradigms such as the emotional attentional blink (EAB), which indexes attentional prioritization of emotional stimuli under conditions of limited processing capacity and may provide a more reliable measure of early‐stage attentional engagement.

Moreover, many studies rely exclusively on self‐reported aggression, limiting conclusions about behavioral responding under provocation. Thus, although attentional bias is positioned as an early encoding mechanism within SIP, its unique contribution to aggressive behavior remains unclear.

Hostile interpretation bias is the tendency to attribute malicious intent in ambiguous situations (Crick and Dodge 1994; Dillon et al. 2016). Within SIP, interpretation is central, as perceived intent shapes anger and justifies retaliation (Dodge et al. 2015). Evidence shows that individuals who interpret ambiguity as hostile are more likely to engage in aggressive and antisocial behavior (De Castro et al. 2002; Martinelli et al. 2018; Verhoef et al. 2019). Small‐to‐moderate associations are observed across development (Gagnon and Rochat 2017), and longitudinal findings indicate hostile interpretations prospectively predict aggression (Dodge et al. 2015).

Compared to attentional bias, hostile interpretation bias shows relatively robust associations with both self‐reported and laboratory‐based aggression (Wilkowski and Robinson 2008), supporting its role as a proximal cognitive mechanism. However, most studies examine interpretation bias independently of earlier encoding processes. If interpretation is shaped by selective attention, studying it in isolation may give an incomplete account of cognitive risk.

SIP proposes that encoding and interpretation are sequential and interdependent (Crick and Dodge 1994). Biased attention to hostile cues may increase threat salience, promoting hostile attributions, while hostile schemas may also guide attention toward threat (Lin et al. 2016). This bidirectional account suggests a self‐reinforcing cycle that may increase aggression risk beyond either bias alone.

Evidence suggests attentional and interpretational biases may not operate independently.

For example, Schippell et al. (2003) found that reduced attention to social threat cues was associated with greater hostile attributions and aggressive behavior, while Horsley et al. (2010) reported that individuals with aggressive tendencies showed altered attentional patterns alongside biases in social memory. These findings highlight complex relationships between early attentional processing and downstream interpretation.

Despite this theoretical integration, empirical tests of the combined contribution of attentional and interpretation biases remain scarce. Most research isolates one cognitive process, leaving unresolved whether these biases represent (a) parallel but independent predictors, (b) partially overlapping mechanisms reflecting a broader hostile‐processing disposition, or (c) synergistic risk factors whose co‐occurrence disproportionately increases aggression. Distinguishing among these possibilities has important implications for aggression theory. Evidence of independent effects would support multiple cognitive pathways within SIP. Evidence of interaction would support cumulative cognitive risk models, indicating that early encoding biases amplify the behavioral consequences of hostile interpretations. Conversely, null interactive effects would suggest more separable mechanisms.

A further limitation of the literature concerns reliance on single‐method assessments. Given reliability concerns surrounding traditional reaction‐time measures (Schmukle 2005), multimethod approaches are needed to more accurately estimate attentional bias effects. Similarly, aggression is multifaceted and not fully captured by self‐report measures. Laboratory paradigms assessing aggression under controlled provocation provide complementary insight into situationally elicited aggressive responding (Anderson and Bushman 2002). Integrating behavioral and dispositional measures allows for more precise evaluation of whether cognitive biases relate to trait aggression, reactive aggression, or both. Notably, aggression elicited under laboratory provocation is typically conceptualized as reactive aggression, which is most strongly linked to early‐stage SIP biases.

The present study examines these processes within a general population adult sample, where effect sizes may be expected to be smaller than in clinical or high‐risk groups. The present study seeks to advance understanding of the cognitive mechanisms underlying aggression by jointly examining attentional bias toward angry faces and hostile interpretation bias, and by testing their independent and interactive associations with aggression. Specifically, three research questions (RQs) are addressed.

The first (RQ1) examines whether attentional bias toward angry faces is associated with self‐reported aggression and antisocial behavior, as well as with behavioral aggression elicited in a laboratory task. Based on prior findings, we hypothesize (H1) a positive association between attentional bias toward angry faces (ATB index), aggression (behavioral and self‐reported), and antisocial outcomes.

The second (RQ2) examines whether hostile interpretation bias is associated with self‐reported aggression, antisocial behavior, and behavioral aggression. Consistent with prior literature, we hypothesize (H2) that hostile interpretation bias will show robust positive associations with aggression (behavioral and self‐reported) and antisocial outcomes.

Finally, the third (RQ3) tests whether attentional bias and hostile interpretation bias interact to predict the outcomes mentioned above. We hypothesize (H3) that individuals exhibiting high levels of both biases will display the greatest levels of aggression and antisocial behavior, consistent with SIP‐based accounts of cumulative cognitive risk.

## 2 Methods

### 2.1 Participants

Eighty‐three participants (*M* = 37.23 years, SD = 11.08, range = 18–72) were recruited from the general population. Sixteen of these participants were excluded: twelve participants showed poor data quality on the EAB paradigm (criteria set as scoring 0 on the ‘Angry or Neutral Only' EAB trials or on ‘the average accuracy across Neutral T1‐Angry T2 and Angry T1‐Neutral T2 conditions') and four participants had incomplete EAB data. The final sample included 67 healthy participants (*M* = 37.43 years, SD = 11.44, range = 18–72). The final sample comprised 36 males (53.7%), 29 females (43.3%), one participant identifying as non‐binary/third gender (1.5%), and one participant preferring not to disclose gender (1.5%). The sample was predominantly White British (68.7%), with additional representation from White European (11.9%), African (6.0%), South Asian (4.5%), and other ethnic backgrounds.

Of these 67 participants, 53 completed the CRTT task and provided usable CRTT data, whereas 14 participants did not complete the CRTT task (i.e., missing CRTT data due to task non‐completion). No additional exclusions were applied to the CRTT data beyond task non‐completion. Consequently, analyses involving CRTT variables are based on the subsample of participants with available CRTT data (*n* = 53), while all other measures use the full sample unless otherwise specified.

The inclusion criteria were set as participants being: (1) aged 18+ years; (2) with normal or corrected‐to‐normal vision; (3) fluent in English language; (4) with no evidence, or history of, mental illness diagnoses, serious neurological or head injury; (5) no current and/or recent substance abuse; and (3) not currently taking prescription drugs and/or supplements that could affect brain function. Participation was based on self‐selection, and no formal screening or verification procedures were conducted.

### 2.2 Design and Procedure

Participants were recruited via opportunistic sampling through an online advertisement on the Testable Minds platform. Demographic data and self‐report measures were collected using Qualtrics. Participants completed measures of trait aggression, hostile interpretation, and antisocial behavior, followed by two experimental tasks.

The study was approved by the College of Health, Medicine and Life Sciences Research Ethics Committee at Brunel University of London (31370‐A‐Dec/2021‐35504‐2). All participants provided online informed consent and received a £10 Amazon voucher for participation.

### 2.3 The Competitive Reaction Time Task

The Competitive Reaction Time Task (CRTT) is a widely used experimental paradigm for assessing aggressive responding in laboratory settings (McCarthy and Elson 2018) and demonstrates good concurrent validity with real‐world aggressive behavior (Warburton and Bushman 2019). While often linked to reactive aggression, the CRTT does not directly distinguish reactive and proactive aggression; instead, it indexes changes in aggressive responding as a function of experimentally manipulated provocation.

**Figure 1:**
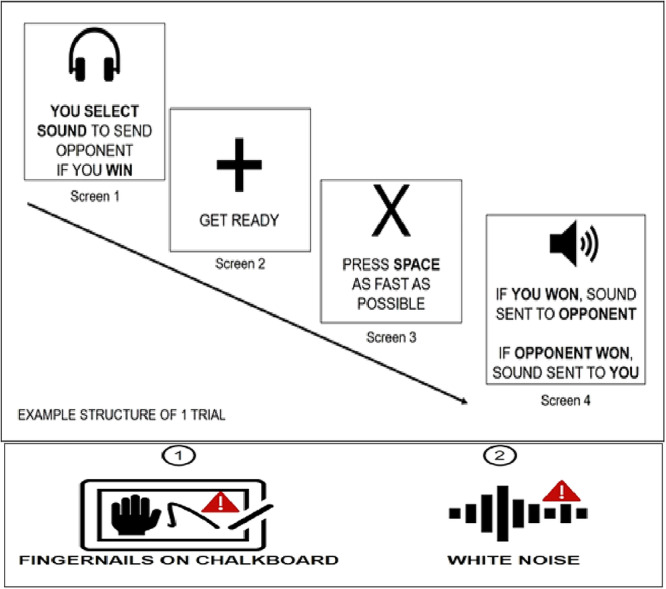
The Competitive Reaction Time Task ‐ example Structure of 1 Trial with Sound Choice. [Color figure can be viewed at wileyonlinelibrary.com]

A similar version of the CRTT was used (Figure 1). Participants believed they were competing against an opponent via a computer server, responding as quickly as possible to a target letter “X” by pressing the spacebar. Before each trial, they selected the volume (65–105 dB; 1–10) and duration (500–5000 ms; 1–10) of an aversive sound (e.g., fingernails on a chalkboard or white noise) to deliver if they won. Higher volume and longer duration indexed greater aggression.

After selection, participants saw the opponent's chosen sound parameters. A fixation cross (500–1500 ms), a random interstimulus interval (500–2500 ms), and the target then followed. Faster responses resulted in a win and delivery of the selected sound; otherwise, the opponent's sound was received.

Provocation was manipulated across trials via systematically increasing intensity of opponent‐administered noise (i.e., combinations of volume and duration), creating low, medium, and high provocation blocks (eight trials each). All participants experienced equal wins and losses across conditions, and sounds were drawn from the International Affective Sounds database.

Behavioral aggression indices were operationalized as the mean selected sound volume and the mean duration within low, medium, and high provocation blocks. These scores were first standardized (*z*‐scored) and then averaged to form a single composite behavioral aggression index for each provocation level (low, medium, high). Higher scores reflected greater aggressive responding as a function of increasing provocation.

### 2.4 The Emotional Attentional Blink

The Emotional Attentional Blink (EAB) paradigm (Raymond et al. 1992) assesses attentional capacity when two targets (emotionally salient T1 and neutral T2) appear in rapid succession within a Rapid Serial Visual Presentation (RSVP) stream. Detecting T1 impairs the detection of T2 when presented 200–500 ms later. This effect varies by emotional salience and lag (number of distractors between T1 and T2): it is reduced when T2 is emotional and prolonged when T1 is emotional (Schwabe et al. 2011), indicating greater attentional demand for emotional stimuli.

**Figure 2:**
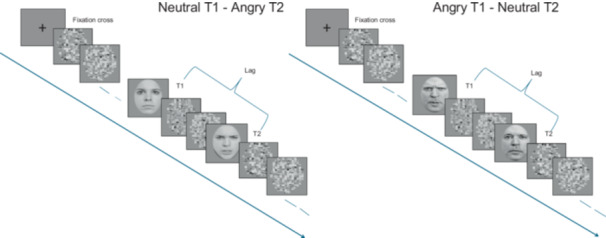
Emotional Attentional Blink **‐** example Structure of 1 Trial. [Color figure can be viewed at wileyonlinelibrary.com]

The task comprised three blocks of 32 trials: Angry T1–Neutral T2, Neutral T1–Angry T2, and single‐target control trials (Neutral or Angry). After three practice trials, 96 trials were presented randomly. Eight lags (70–560 ms) were used, with four trials per lag. T1 appeared at positions 8–11; in single‐target trials, the target appeared at positions 9–16. Following Müsch et al. (2012), each trial began with a 500 ms fixation, then a 70 ms stream of 25 stimuli (target faces and scrambled distractors) (see Figure 2). Participants reported whether they saw two faces (Yes/No) and the emotional expression(s) (Angry/Neutral); the second question was omitted if only one face was reported. The task lasted ~12 min. Attentional blinks were calculated per lag as correct T1 identification with incorrect or absent T2 identification.

### 2.5 Attentional Bias to Threat: Rationale and Computation of the Attentional Bias (ATB) Index

Aggressive individuals often show heightened attention to threat, especially angry faces, but traditional measures of this bias are unreliable. Using the EAB described above offers a more controlled way to test whether angry faces capture attention more strongly and whether they are more likely to “break through” limited attentional capacity. If angry faces have attentional priority, participants should detect Angry T2 faces more accurately than Neutral T2 faces during the blink (Lag 3–8). To quantify this threat‐related prioritisation, we computed an ATB index, capturing the relative detectability of Angry T2 versus Neutral T2 faces under different emotional T1 conditions.

Two key conditions reflect strong bias *towards* angry faces:

1. NeutralT1–AngryT2:
2. Higher T2 accuracy here indicates that angry faces are more likely to break through the blink when T1 was neutral.
3. AngryT1–NeutralT2:
4. Lower T2 accuracy here indicates that processing an angry T1 consumes attentional resources, impairing awareness of a Neutral T2 that follows.

To ensure the index reflects performance during the attentional blink, only Lag 3–8 (210–560 ms) are used. Early lags (Lag 1–2) are excluded due to extremely low detectability regardless of emotion.

The ATB index is calculated by subtracting the mean accuracy for trials with an Angry T1 followed by a Neutral T2 from the mean accuracy for trials with a Neutral T1 followed by an Angry T2. In formula form: ATB = (Accuracy: Neutral T1–Angry T2) – (Accuracy: Angry T1–Neutral T2). Positive scores indicate a bias toward angry faces, a score of zero reflects no bias, and negative scores indicate a bias toward neutral faces.

### 2.6 Trait Aggression

Trait aggression was assessed using the 29‐item Buss–Perry Aggression Questionnaire (AQ; Buss and Perry 1992), comprising four subscales: Physical Aggression, Verbal Aggression, Anger, and Hostility. Items were rated on a 5‐point Likert scale (1 = ‘extremely uncharacteristic,’ 5 = ‘extremely characteristic’), with two items reverse‐scored. Higher scores indicate greater trait aggression. The AQ shows good reliability (Hasliza et al. 2016; Christopher et al. 2024). In this sample, Cronbach's α values were: Physical = 0.88, Verbal = 0.76, Anger = 0.91, Hostility = 0.88, Total = 0.94.

### 2.7 Hostile Interpretation

Hostile attribution bias was assessed using the Word Sentence Association Paradigm for Hostility (WSAP–H; Dillon et al. 2016). Twenty ambiguous sentences were paired with either a hostile (e.g., ‘Inconsiderate’) or benign (e.g., ‘Accidental’) word. Participants rated the match on a 6‐point Likert scale, with each scenario presented twice (hostile and benign). Subscale scores were averaged, and an interpretation bias score was calculated by subtracting benign from hostile ratings (Kuckertz et al. 2013), with higher scores indicating greater hostility. Cronbach's *α* was 0.76 (Hostile) and 0.85 (Benign). Both indices were retained for correlational analyses for completeness. However, the Hostile interpretation score was used as the primary outcome in regression analyses, as it reflects absolute hostile interpretation bias and is consistent with prior WSAP research (Dillon et al. 2016).

### 2.8 Antisocial Behavior

Antisocial behavior was assessed using the 32‐item Sub‐Types of Antisocial Behavior questionnaire (STAB; Burt and Donnellan 2009), comprising Physical, Social, and Rule‐Breaking subscales. Items were rated on a 5‐point Likert scale (1 = ‘*Never*,’ 5 = ‘*Nearly all the time*’). A total score was computed by summing subscales, with higher scores indicating greater antisocial behavior. The STAB shows good reliability (Burt and Donnellan 2010). In this sample, Cronbach's *α* values were 0.93 across all subscales.

### 2.9 Power Analysis

An a priori power analysis conducted using G*Power 3.1 indicated that, for a regression with three predictors (ATB index, hostile interpretation bias, and their interaction), a sample of *N* = 55 is required to detect a medium‐sized interaction (*f* ^2^ = 0.15) at *α* = 0.05% and 80% power. The present sample (*N* = 67) was therefore adequately powered to detect medium‐sized interaction effects, consistent with expectations for aggression paradigms.

### 2.10 Statistical Analysis

Analyses were conducted in SPSS 29 (SPSS Inc., Chicago, IL) with *α* = 0.05. CRTT provocation effects were tested using paired‐samples t‐tests comparing low‐ and high‐provocation conditions on noise volume and duration. The EAB was analysed using a 2 (Condition: Neutral T1–Angry T2 vs. Angry T1–Neutral T2) × 8 (Lag: 1–8) repeated‐measures ANOVA on attentional blink counts.

Hypotheses were tested as follows: H1, Pearson correlations between ATB and aggression/antisocial outcomes; H2, Pearson correlations between WSAP hostile scores and outcomes; H3, multiple regression analyses testing whether ATB, hostile interpretation, and their interaction predicted aggression and antisocial behavior. All predictors were standardized (z‐scored) prior to analysis, and interaction terms were created by multiplying standardized variables to reduce multicollinearity and follow recommended moderation procedures.

## 3 Results

### 3.1 Manipulation Check CRTT

Confirming the effectiveness of the provocation manipulation, participants' mean volume of aggression response was higher in the high‐provocation condition (*M* = 3.89, SD = 2.59) than the low‐provocation condition (*M* = 3.49, SD = 2.30). This difference approached significance in the predicted direction, *t*(52) = −1.60, *p* = 0.058, with a small effect size, Cohen's *d* = −0.22. Mean duration of aggressive responses was also greater under high provocation (*M* = 3.79, SD = 2.60) than low provocation (*M* = 3.27, SD = 2.23), *t*(52) = −1.95, *p* = 0.028, with a small‐to‐moderate effect size, Cohen's *d* = −0.27. However, this pattern indicates a mixed manipulation check outcome, with stronger and more reliable effects observed for duration than for volume, suggesting some variability in the robustness of provocation effects across outcome measures.

### 3.2 Manipulation Check Attentional Blink and Emotional Modulation of Attention

Confirming the expected temporal constraints of attention in the EAB task, there was a significant main effect of Lag, F(5.88, 388.29) = 17.68, *p* < 0.001, *η* ^2^_p_ = 0.211, indicating a robust attentional blink. Blink rates were highest at early lags (Lags 1–3), where T1–T2 temporal proximity was greatest, and decreased across later lags, consistent with reduced processing difficulty as temporal separation increased.

**Figure 3:**
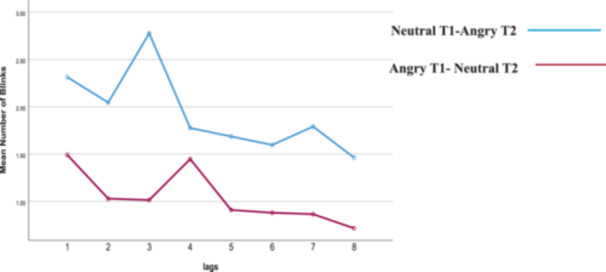
Mean Number of Attentional Blinks Across Lags by Condition. Note. Mean number of attentional blinks across temporal lags for neutral and angry stimuli at T1 and T2. Error bars indicate +/−1SE. [Color figure can be viewed at wileyonlinelibrary.com]

There was also a significant main effect of Condition, F(1, 66) = 40.21, *p* < 0.001, *η* ^2^_p_ = 0.379, with more blinks in the Neutral T1→Angry T2 condition than the Angry T1→Neutral T2 condition, confirming emotional modulation of attention. A significant Condition × Lag interaction, F(5.65, 372.72) = 8.85, *p* < 0.001, *η* ^2^_p_ = 0.118, indicated that effects varied across the temporal window of the AB. Differences were strongest at Lag 3 ( ≈ 210–300 ms post‐T1), consistent with peak AB sensitivity and theories of limited attentional resources for emotional stimuli. Overall, results confirm a canonical attentional blink and its modulation by emotional content (Figure 3).

### 3.3 Is Attentional Bias Toward Angry Faces (ATB Index) Associated With Antisocial Behavior, Self‐Report and Behavioral Aggression (H1)?

A small but significant positive correlation was found between ATB index and the behavioral aggression composite, *r*(51) = 0.29, *p* = 0.038, indicating that greater attentional bias toward angry faces was associated with higher behavioral aggression. No significant correlations were observed between ATB and AQ self‐reported aggression (*r*(65) = –0.16, *p* = 0.190) or STAB antisocial behavior (*r*(65) = –0.21, *p* = 0.096).

The regression model predicting behavioral aggression from ATB was significant, F(1, 51) = 4.56, *p* = 0.038, explaining 8.2% of the variance (*R* ^2^ = 0.08). ATB was a significant positive predictor (*β* = 0.29, t(51) = 2.14, *p* = 0.038), with higher bias scores associated with increased behavioral aggression.

Overall, ATB was associated with behavioral but not self‐reported aggression or antisocial behavior.

### 3.4 Is Hostile Interpretation Associated With Aggression (Self‐Report and Behavioral) and Antisocial Behavior (H2)?

Hostile interpretation (WSAP Hostile) was significantly associated with AQ self‐reported aggression, *r*(65) = 0.36, *p* = 0.003, and STAB antisocial behavior, *r*(65) = 0.28, *p* = 0.021. The WSAP hostility bias score showed a similar pattern with AQ, *r*(65) = 0.32, *p* = 0.008, but was not significantly related to STAB, *r*(65) = 0.09, *p* = 0.451. Neither WSAP measure correlated with the behavioral aggression composite, *r*(51) ≤ 0.15, *p* ≥ 0.300.

Regression analyses further supported these associations. Hostile interpretation significantly predicted antisocial behavior, F(1, 65) = 5.57, *p* = 0.021, explaining 7.9% of the variance (*R* ^2^ = 0.08), with WSAP Hostile as a significant predictor, *β* = 0.28, t(65) = 2.36, *p* = 0.021. It also significantly predicted AQ aggression, F(1, 65) = 9.40, *p* = 0.003, accounting for 12.6% of the variance (*R* ^2^ = 0.13), with WSAP Hostile again a significant predictor, *β* = 0.36, *t*(65) = 3.07, *p* = 0.003.

Overall, a hostile interpretation was associated with self‐reported aggression and antisocial behavior, but not behavioral aggression.

### 3.5 Interaction: Do ATB Index and Hostile Interpretation Score Interact to Predict Aggression (Self‐Report and Behavioral) and Antisocial Behavior (H3)?

#### 3.5.1 Predicting Antisocial Behavior Tendencies (STAB_TOTAL)

A multiple regression model, examining whether ATB index, hostile interpretation, and their interaction predicted antisocial behavior tendencies, was statistically significant, *F*(3, 63) = 2.91, *p* = 0.041, explaining 12.2% of the variance (*R^2^* = 0.12). Hostile interpretation significantly predicted higher antisocial behavior tendencies, *b* = 0.16, *SE* = 0.07, *t* = 2.28, *p* = 0.026. ATB index was not a significant predictor, *b* = −0.11, *SE* = 0.07, *t* = −1.58, *p* = 0.119. The interaction between ATB index and hostile interpretation was also not significant, *b* = −0.04, *SE* = 0.08, *t* = −0.46, *p* = 0.645 (Table 1). Thus, hostile interpretation uniquely predicted antisocial behavior tendencies, whereas the ATB index and the interaction term did not contribute additional explanatory power.

**Table 1:** Regression models predicting antisocial behavior, trait aggression, and task‐induced aggression (under high and low provocation) from attentional bias, hostile interpretation, and their interaction.

Outcome/Predictor	*B*	SE B	*β*	*t*	*p*
**Antisocial Behavior (STAB_TOTAL)**					
Constant	10.34	1.87	—	5.53	< 0.001
Attentional Bias (ATB)	−0.11	0.07	−0.17	−1.58	0.119
Hostile Interpretation (WSAP)	0.16	0.07	0.28	2.28	0.026*
ATB × Hostile Interpretation	−0.04	0.08	−0.04	−0.46	0.645
**Trait Aggression (AQ_Total)**					
Constant	20.12	3.12	—	6.45	< 0.001
Attentional Bias (ATB)	−3.88	2.39	−0.20	−1.62	0.110
Hostile Interpretation (WSAP)	7.67	2.36	0.36	3.24	0.002**
ATB × Hostile Interpretation	4.39	2.82	0.17	1.56	0.124
**Task‐Induced Aggression: High Provocation**					
Constant	5.12	1.34	—	3.82	< 0.001
Attentional Bias (ATB)	0.38	0.21	0.21	1.78	0.080
Hostile Interpretation (WSAP)	0.15	0.20	0.09	0.46	0.645
ATB × Hostile Interpretation	0.62	0.22	0.37	2.85	0.006**
**Task‐Induced Aggression: Low Provocation**					
Constant	2.84	1.22	—	2.33	0.024
Attentional Bias (ATB)	0.25	0.18	0.18	1.25	0.217
Hostile Interpretation (WSAP)	0.01	0.19	0.01	0.06	0.949
ATB × Hostile Interpretation	0.33	0.23	0.20	1.40	0.168

*Note:* *p < 0.05 and **p < 0.01.

#### 3.5.2 Predicting Trait Aggression (AQ_Total)

A second regression model, assessing whether ATB index, hostile interpretation accounted for variance in trait aggression, was also statistically significant, *F*(3, 63) = 4.67, *p* = 0.005, explaining 18.2% of the variance (*R^2^* = 0.18). Hostile interpretations significantly predicted higher trait aggression, *b* = 7.67, *SE* = 2.36, *t* = 3.24, *p* = 0.002. ATB index was not a significant predictor, *b* = −3.88, *SE* = 2.39, *t* = −1.62, *p* = 0.110. The interaction between ATB index and hostile interpretations was also not significant, *b* = 4.39, *SE* = 2.82, *t* = 1.56, *p* = 0.124 (Table 1). Thus, hostile interpretations again emerged as a robust predictor of trait aggression, independent of ATB index.

#### 3.5.3 Predicting Task‐Induced Aggression Under High Provocation (CRTT)

**Figure 4:**
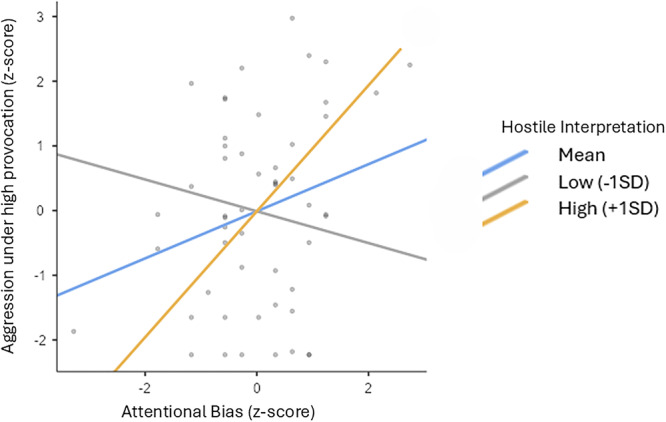
Interaction between attentional bias (ATB) and interpretation bias (WSAP‐hostile) predicting aggression. [Color figure can be viewed at wileyonlinelibrary.com]

A third regression model, assessing whether ATB index, hostile interpretation predicted aggressive behavior under high provocation (Aggression Composite_Vol_Dur_High) was statistically significant, *F*(3, 49) = 4.54, *p* = 0.007, accounting for 21.8% of the variance (*R^2^* = 0.22). The interaction between ATB index and hostile interpretation significantly predicted aggressive behavior under high provocation, *b* = 0.62, *SE* = 0.22, *t* = 2.85, *p* = 0.006 (Table 1). Attentional bias alone was not significant (*p* = 0.098), nor were hostile interpretations alone (*p* = 0.340). To probe the interaction, simple slopes analyses were conducted. Attentional bias was not significantly associated with aggression at low levels of hostile interpretation ( − 1 SD), *b* = −0.25, SE = 0.30, *z* = −0.82, *p* = 0.413. However, attentional bias significantly predicted aggression at mean levels of hostile interpretation, *b* = 0.36, SE = 0.17, *z* = 2.13, *p* = 0.034, and more strongly at high levels ( + 1 SD), *b* = 0.97, SE = 0.27, *z* = 3.67, *p* < 0.001 (see Figure 4).

These findings indicate that task‐induced aggression under high provocation is most strongly associated with attentional bias in the context of elevated hostile interpretation, consistent with a potentiation effect.

Furthermore, to examine the robustness of the observed moderation effect, we repeated the analysis separately for each behavioral index of aggression (noise volume and noise duration). The interaction between attentional bias and hostile interpretation was significant for both noise volume (*β* = 0.443, *p* = 0.002) and noise duration (*β* = 0.350, *p* = 0.014). In both cases, simple slope analyses showed that attentional bias was positively associated with aggressive responding under high hostile interpretation but not under low hostile interpretation, indicating a consistent moderation pattern across behavioral outcomes.

Crucially the same analysis was performed for the outcome aggressive behavior under low provocation and it was found that neither ATB index or hostile interpretation or their interaction significantly predicted aggressive behavior under low provocation, *F*(3, 49) = 1.47, *p* = 0.235, explaining only 8.3% of the variance (*R* ^2^ = 0.08); ATB index, *b* = 0.25, *p* = 0.217; hostile interpretations, *b* = 0.01, *p* = 0.949; interaction term, b = 0.33, *p* = 0.168.

### 3.6 False Discovery Rate (FDR) Correction Across Analyses

To address multiple comparisons, a false discovery rate (FDR; Benjamini–Hochberg) correction was applied separately within each hypothesis set (H1–H3). For H1 (attentional bias and aggression outcomes), the association with behavioral aggression did not survive correction and should be interpreted cautiously. For H2 (hostile interpretation and aggression outcomes), associations with AQ and STAB remained statistically significant after correction. For H3 (interaction effects), interactions predicting behavioral aggression under provocation (CRTT composite, noise volume, and duration) remained significant, whereas those for self‐report outcomes did not.

Overall, robust effects were observed for hostile interpretation main effects and the interaction between attentional and interpretative biases in predicting behavioral aggression under provocation.

## 4 Discussion

The present study examined the independent and interactive contributions of attentional bias toward angry faces and hostile interpretation bias in predicting antisocial behavior and aggression, using behavioral and self‐report measures. Guided by Social Information Processing (SIP) theory (Crick and Dodge 1994), we tested whether early (attentional) and later (interpretative) biases uniquely and jointly predict aggression under varying provocation. Three findings emerged. First, hostile interpretation bias predicted dispositional aggression and antisocial tendencies. Second, attentional bias showed a modest association with behavioral aggression under provocation but not self‐report. Third, attentional and interpretative biases interacted to predict aggression under high provocation. Overall, cognitive biases contribute to aggression in a stage‐specific and context‐dependent manner.

Consistent with our first hypothesis (partially supported), attentional bias toward angry faces was associated with behavioral aggression in the CRTT, but not with self‐reported aggression or antisocial tendencies. This aligns with prior research reporting small and inconsistent associations between attentional bias and trait aggression (Cooper and Langton 2006; Wilkowski et al. 2015). The findings suggest attentional bias may be more relevant for reactive behavior under provocation than dispositional self‐report.

However, this finding should be interpreted cautiously, given multiple testing. After FDR correction across H1 analyses, the association between attentional bias and behavioral aggression was no longer significant, indicating a modest effect sensitive to sampling variability and requiring replication in larger samples. In contrast, all significant effects in H2 and H3 survived FDR correction.

Theoretically, attentional biases reflect early encoding processes that prioritize threat‐relevant stimuli (Fox et al. 2001; Yiend 2010). Within dual‐process models of aggression (Anderson and Bushman 2002), these mechanisms are automatic and stimulus‐driven, shaping early affective processing and biasing subsequent behavioral decision‐making. Attentional capture by angry faces may therefore heighten threat sensitivity and arousal, which can influence controlled and intentional aggressive responding when provocation is salient in laboratory paradigms such as the CRTT. However, because these processes operate rapidly and often outside awareness, they may not align with stable self‐perceptions of aggressiveness measured via questionnaires.

Methodologically, the emotional attentional blink (EAB) paradigm strengthens this interpretation. Reaction‐time difference scores from dot‐probe tasks have been criticized for limited reliability (Kappenman et al. 2014; Schmukle 2005). The EAB indexes temporal prioritization of emotional stimuli under attentional load and may provide a more robust measure of threat‐related engagement. Its association with behavioral aggression suggests that attentional blink–based indices may better capture mechanisms relevant to reactive aggression.

Consistent with our second hypothesis, hostile interpretation bias showed reliable associations with self‐reported aggression and antisocial behavior, converging with evidence linking hostile attribution tendencies to aggression (De Castro et al. 2002; Dodge and Pettit 2003; Wilkowski and Robinson 2008). Importantly, it predicted dispositional aggression even when controlling for attentional bias, underscoring its independent contribution.

In contrast, hostile interpretation bias was not associated with behavioral aggression in the CRTT, likely reflecting differences in cognitive processing stages.

This dissociation likely reflects differences in cognitive processing stages. Interpretation bias involves higher‐order inferential processes through which ambiguous social cues are assigned hostile meaning, contributing to enduring schemas regarding others' intentions. Such schemas are more directly reflected in trait‐based measures. By comparison, behavioral aggression under experimental provocation may depend more strongly on immediate attentional and affective activation than on interpretative schemas in isolation.

These findings reinforce the centrality of interpretative processes within SIP theory (Crick and Dodge 1994). Whereas attentional bias reflects early encoding of threat cues, hostile interpretation bias corresponds to later‐stage meaning‐making processes that shape behavioral scripts and expectations. The present results suggest that these later processes are more closely tied to stable aggressive tendencies.

The key finding was the interaction between attentional and interpretative biases in predicting behavioral aggression under high provocation. Individuals high in both biases showed disproportionately elevated aggression when provocation was salient, with no comparable effect for self‐reported outcomes.

This supports SIP models proposing that distortions across processing stages cumulatively increase aggression risk (Crick and Dodge 1994). Under high provocation, early attentional bias to threat may amplify hostile interpretations, increase anger, and reduce inhibition, raising reactive aggression. When the threat is low, these biases may remain behaviorally inactive. This pattern aligns with person × situation frameworks, where cognitive vulnerabilities require contextual activation.

Prior research shows attentional biases toward social threat are associated with hostile intent attributions (Miller and Johnston 2019), suggesting functional links between early attentional and later interpretative processes. The present findings extend this by showing that these biases interact to predict aggression under heightened provocation. Direct behavioral tests of such multi‐stage interactions remain limited, highlighting the contribution of this study.

These findings refine cognitive models of aggression in several ways. First, attentional and interpretation biases are functionally distinct rather than redundant. Second, their joint influence is conditional on situational provocation and outcome modality, supporting a conditional cumulative risk model. Third, integrating behavioral and self‐report outcomes aligns stage‐based theories with modality‐specific assessment.

Practically, the robust link between hostile interpretation bias and dispositional aggression suggests that individuals who habitually interpret ambiguity as hostile may be at elevated risk of aggressive and antisocial behavior (Crick and Dodge 1994). Interventions targeting attributional biases may therefore reduce trait‐level aggression. Interaction findings further indicate that high provocation may amplify the behavioral effects of combined cognitive vulnerabilities. However, these implications should be interpreted cautiously, given the non‐clinical adult sample. It remains unclear whether similar patterns would emerge in adolescents or clinical populations, or in applied settings such as schools or correctional contexts.

Adolescence is characterized by heightened emotional reactivity and sensitivity to social threat (van den Bos and Hertwig 2017; Tymula et al. 2012), which may increase the impact of cognitive biases on aggression. Future research should test whether the observed interaction generalizes to younger samples and real‐world high‐provocation environments.

Several limitations warrant consideration. The sample was heterogeneous in age and gender, which may introduce variability in cognitive processing and aggression‐related tendencies. Given known differences across gender and lifespan, these factors may have influenced observed associations. Although age and gender were not included as covariates to preserve power and maintain a within‐sample cognitive focus, future research with larger samples should test demographic moderation.

A further limitation is the absence of a reliability estimate for the attentional bias (ATB) index from the emotional attentional blink (EAB) task. Future work should address this, given concerns about attentional bias reliability. The cross‐sectional design also precludes causal inference, requiring longitudinal or experimental approaches to establish directionality. Although powered for main effects, the sample may have lacked sensitivity for small interactions. Finally, while the CRTT is widely used as a laboratory analog of aggression, its ecological validity remains debated.

Future research should examine whether similar interactive effects emerge in clinical or forensic populations and whether interventions targeting both attentional and interpretative processes produce additive or synergistic reductions in aggression.

Hostile interpretation bias robustly predicts dispositional aggression and antisocial behavior, whereas attentional bias toward angry faces is selectively associated with behavioral aggression under provocation. Critically, these biases interact to predict aggression in high‐provocation contexts, supporting SIP‐based models of cumulative cognitive risk (Crick and Dodge 1994). By delineating stage‐specific and context‐dependent mechanisms, these findings advance theoretical understanding of aggression and inform integrative intervention approaches.

## Data Availability

The data that support the findings will be made openly available at the University of Reading data archive.
